# Immunomodulatory effects of two recombinant arginine kinases in *Sarcoptes Scabiei* on host peripheral blood mononuclear cells

**DOI:** 10.3389/fimmu.2022.1035729

**Published:** 2022-11-14

**Authors:** Yanting Xu, Ziyi Xu, Xiaobin Gu, Yue Xie, Ran He, Jing Xu, Bo Jing, Xuerong Peng, Guangyou Yang

**Affiliations:** ^1^ Department of Parasitology, College of Veterinary Medicine, Sichuan Agricultural University, Wenjiang, China; ^2^ Department of Chemistry, College of Life and Basic Science, Sichuan Agricultural University, Wenjiang, China

**Keywords:** *Sarcoptes scabiei*, arginine kinase, PBMC, NF-κB, Th1/Th2, inflammatory

## Abstract

**Background:**

As an important zoonotic parasitic disease with global distribution, scabies causes serious public health and economic problems. Arginine kinase (AK) is involved in cell signal transduction, inflammation, and apoptosis. Two AKs were identified in *Sarcoptes scabiei*, but their functions in the host immune response remain unclear.

**Methods:**

rSsAK-1 and rSsAK-2 were expressed, purified, and immunolocalized. The effects of rSsAK-1 and rSsAK-2 on rabbit PBMC proliferation, apoptosis, and migration; Bcl-2, Bcl-xl, Fas, Bax, and NF-κB transcription levels; and IL-2, IFN-γ, IL-4, IL-10, TGF-β1, and IL-17 secretion were detected.

**Results:**

rSsAK-1 and rSsAK-2 were cloned and expressed successfully. Both enzymes were ~57 kDa and contained 17-kDa tagged proteins, and had good catalytic activity and immunoreactivity. The proteins were located in the *S. scabiei* exoskeleton, chewing mouthparts, legs, stomach, and intestine. SsAK-1 and SsAK-2 were secreted in the pool and epidermis of the skin lesions, which may be involved in *S. scabiei*–host interaction. rSsAK-1 and rSsAK-2 significantly promoted cell proliferation, induced cell migration, inhibited apoptosis, and increased Bcl-2, Bcl-xl and NF-κB (p65) transcription levels concentration-dependently, and inhibited IL-2, IFN-γ, and IL-10 secretion and promoted IL-4 and IL-17 secretion.

**Conclusion:**

rSsAK-1 and rSsAK-2 might increase Bcl-2 and Bcl-xl expression by activating the NF-κB signaling pathway to promote cell proliferation and inhibit apoptosis, which induced PBMC survival. By inducing PBMC migration to the infection site, rSsAK-1 and rSsAK-2 shifted the Th1/Th2 balance toward Th2 and changed the Th17/Treg balance, which indicated their immune role in *S. scabiei* allergic inflammation.

## Introduction

*Sarcoptes scabiei* is a widely distributed ectoparasite that infects domestic animals, wild animals, and humans. So far, there are 149 host species, and the geographical distribution and the number of parasitic host species demonstrated a continuous growth trend (1). Living in the epidermis, *S. scabiei* causes scabies, a persistent, contact, and contagious skin disease characterized by severe itching, crusting, alopecia, and epidermal thickening (1, 2), which may result in secondary bacterial infection causing cellulitis, pyoderma, acute glomerulitis, and rheumatic heart disease (3, 4). Due to co-evolution with its hosts, *S. scabiei* can adjust to the host immune function (5). When burrowing into the skin, *S. scabiei* releases antigens and soluble substances with pharmacological activity (excretory and secretory products) to induce cell responses in tissues and regulate the host inflammatory and immune responses (6). Scabies affects approximately 300 million people worldwide every year and the infection and incidence rates are more obvious in developing countries (especially poor areas) (6).

Belonging to the phosphagen kinase family involved in cell signal transduction, metabolism, inflammation, apoptosis and autophagy, arginine kinases (AKs) are mainly distributed in invertebrates (arthropods, mollusks, protozoa), and catalyze the reaction between MgATP and L-arginine during energy demand (7–9). AKs are involved in antioxidative stress and the innate immune responses and directly regulate the balance of the ATP energy pool and participate in cellular energy metabolism (10). Liquid chromatography/tandem mass spectrometry (LC-MS/MS) revealed that two AK family genes in the *S. scabiei* genome (SsAK-1 and SsAK-2, respectively) encoded excretory proteins, but their functions are unknown (11–13).

Here, we determined the prokaryotic expression and distribution of SsAK-1 and SsAK-2. We stimulated rabbit peripheral blood mononuclear cells (PBMCs) with recombinant SsAK-1 and SsAK-2 (rSsAK-1 and rSsAK-2, respectively) to determine their effects on cell proliferation, apoptosis, and migration and the transcription levels of apoptosis-related factors (Bcl-2, Bcl-xl, Fas, Bax) and NF-κB to explore the role of SsAK-1 and SsAK-2 on cell survival and migration to inflammation sites. Furthermore, we detected the effects of rSsAK-1 and rSsAK-2 on IL-2, IFN-γ, IL-4, IL-10, TGF-β1, and IL-17 secretion to investigate the effect of rSsAK-1 and rSsAK-2 on Th1/Th2 and Th17/Treg balance. The findings present evidence for understanding the role of rSsAK-1 and rSsAK-2 in parasite–host interactions and elucidating the pathogenic mechanism of scabies to provide a reference for scabies clinical treatment.

## Materials and methods

### Ethics approval and consent to participate

This animal study was reviewed and approved by the Sichuan Agricultural University Animal Ethics Committee. All procedures related to experimental animals were strictly implemented in accordance with the Guidelines for the Care of Laboratory Animals of the Sichuan Agricultural University Animal Ethics Committee (SYXK 2019–189). The animal procedures used in this study were performed in accordance with the recommendations of the Guide for the Care and Use of Laboratory Animals (National Research Council, Bethesda, MD, USA) and the ARRIVE guidelines. All methods were performed in accordance with relevant guidelines and regulations.

### Animals, mite, and serum

Six female Sprague-Dawley rats (specific pathogen-free, 6 weeks old, 150–200 g) were purchased from SPF (Beijing) Biotechnology Co., Ltd. Six-month-old healthy New Zealand rabbits (three males and three females, 3–3.25 kg) were purchased from Chengdu Dashuo Laboratory Animal Co., Ltd. *S. scabiei* mites and positive serum were collected from scabies-infected rabbits provided by the Department of Parasitology, College of Veterinary Medicine, Sichuan Agricultural University. Negative serum was collected from healthy rabbits provided by a rabbit farm that had not experienced scabies mite infestation.

### Preparation of rSsAKs

The SsAK-1 (GenBank accession number JXLN01011522.1) and SsAK-2 genes (GenBank accession number OP185092) were screened according to the scabies mite transcriptome and genome databases reported in the National Center for Biotechnology Information. Specific primers with restriction sites for amplification were designed using Primer Premier 5.0 (**Table 1**). SsAK-1 and SsAK-2 were amplified as follows: 94°C for 3 min, followed by 35 cycles of amplification at 94°C for 30 s, 55°C for 45 s, 72°C for 1 min, and 72°C for 10 min. The amplified fragments were individually linked with pET-32a(+) vector (Invitrogen) and transfected into *Escherichia coli* BL21 cells. Protein expression was examined with isopropyl-β-D-thiogalactopyranoside (IPTG) for 12 h at 22°C. The rSsAKs were purified by nickel chelate affinity chromatography columns and detected by 12% sodium dodecyl sulfate–polyacrylamide gel electrophoresis (SDS-PAGE). The rSsAK concentrations were detected by bicinchoninic acid assay (Takara Bio) and their activity was analyzed by phosphorus determination.

**Table 1:** Restriction primer sequences of the SsAK-1 and SsAK-2 genes.

Gene	Restriction primer sequence	Restriction site
SsAK-1	F:5’-CGGGATCCATGGTTGATCAAGCTGTTATG-3’	BamHI
	R:5’-CGAGCTCTTACAATGATTTTTCGATCTTG-3’	SacI
SsAK-2	F:5’- CGGGATCCATGGTCGATCAAGCAGTTATC-3’	BamHI
	R:5’- CGAGCTCTTACATCGATTTCTCAATCTTAAT-3’	SacI

### Sequence analysis

The molecular weights and isoelectric points (pI) of the rSsAKs were predicted by ExPASy (http://web.expasy.org/protparam/). Whether the proteins had a signal peptide and a transmembrane region were predicted with SignalP 4.0 and the TMHMM 2.0 server (http://www.cbs.dtu.dk/services/TMHMM-2.0/) (14). Multiple sequence alignment and secondary structure were analyzed with Jalview 2.10.5. MEGA 5.05 neighbor-joining (NJ) was used to perform phylogenetic analysis and construct an evolutionary tree.

### Western blotting

Total bacterial extract and purified rSsAKs were separated by 12% SDS-PAGE. The western blotting analysis was performed as previously described (14). The primary antibody was rabbit serum or rat serum (diluted 1:150 with 0.01 mol/L phosphate-buffered saline [PBS]). The secondary antibody was horseradish peroxidase (HRP)-conjugated goat anti-rabbit/rat antibody (diluted 1:2000) (Absin, Shanghai, China).

### Hematoxylin-eosin staining and immunolocalization

Observation of skin tissue morphology using H&E staining and the immunolocalization of rSsAK-1 and rSsAK-2 in mites and skin was performed as previously described (14). The primary antibody was rat serum (diluted 1:150 with 0.01 mol/L PBS) and the secondary antibody was fluorescein isothiocyanate (FITC) goat anti-rat IgG (diluted 1:200) (Abclonal, Wuhan, China).

### PBMC separation and rSsAK processing

Aseptic blood was collected from the marginal ear vein of the rabbits. The rabbit PBMCs were isolated by an animal PBMC isolation kit (Solarbio) and resuspended in RPMI 1640 medium (Solarbio) containing 10% fetal bovine serum (Sigma), penicillin (100 U/mL), and streptomycin (0.1 mg/mL) (Gibco). Trypan blue staining was used to ensure that the cell viability was >95%. Endotoxins were removed from the rSsAKs by an endotoxin removal kit (Smart-Lifesciences).

### Cell proliferation assay

PBS (0.01 mol/L, control), pET-32a (tagged protein), concanavalin A (ConA, 10 µg/mL), and rSsAKs (10, 20, 40, 80 µg/mL) were co-cultivated with 100 µL cell suspension (1 × 10^6^/mL) in a 37°C 5% CO_2_ incubator for 24 h. After 1-h incubation with 10 µL Cell Counting Kit-8 (CCK-8, Beyotime Biotechnology), the absorbance of the samples was measured at 450 nm.

### Apoptosis assay

PBS, pET-32a, and rSsAKs (10, 20, 40, 80 µg/mL) were co-cultivated with 1 mL cell suspension (1 × 10^6^/mL) in a 37°C 5% CO_2_ incubator for 24 h. The cells were mixed gently and transferred to a centrifuge tube for 8-min centrifugation at 2000 rpm, then resuspended in 500 µL PBS. The cells (100 µL) were mixed with 200 µL 1× binding buffer. Next, 5 µL annexin V–FITC and 10 µL propidium iodide (PI) were added in succession to stain the cells. Finally, the cells were resuspended in 400 µL PBS and analyzed by flow cytometry (Backman).

### qRT-PCR analysis of apoptotic factors and NF-κB (p65)

PBS, pET-32a, and rSsAKs (10, 20, 40, 80 µg/mL) were co-cultivated with 1 mL cell suspension (1 × 10^7^/mL) in a 37°C 5% CO_2_ incubator for 24 h. Total cellular RNA was extracted with a total cellular RNA extraction kit (FORE GENE) and the complementary DNA (cDNA) was reverse-transcribed. The GAPDH, β-actin, and 18S genes were preliminarily screened, and the 18S gene was selected as the internal reference gene. qRT-PCR was performed to detect the relative transcription levels of Fas, Bax, Bcl-2, Bcl-xl, and NF-κB (p65) with specific primer sequences (**Table 2**) in a LightCycler 480 System (Roche). The amplification mixture was as follows: 10 µL TB Green Premix Ex Taq II, 0.8 µL forward and reverse primers, 2 µL cDNA, and 6.4 µL double-distilled water. The reaction system was as follows: 95°C for 30 s, followed by 40 cycles of 95°C for 5 s, 62°C for 30 s, 95°C for 5 s, 60°C for 1 min, and 50°C for 30 s. The results were analyzed by the comparative threshold cycle method (2^-ΔΔCt^).

**Table 2:** Primer sequences of the Fas, Bax, Bcl-2, Bcl-xl, NF-κB (p65), and 18S genes.

Gene	Primer sequence
Fas	F: 5’-TCATTGAGGAATGCACACAAAC-3’
	R: 5’-TGTATCTTCTCAGCCCCAAAAC-3’
Bax	F:5’- CCTTTTGCTTCAGGGTTTCA-3’
	R: 5’-ATCCTCTGCAGCTCCATGTT-3’
Bcl-2	F: 5’-CCCACCCCTGGCATCTTC-3’
	R: 5’-GCGACGGTAGCGACGAGA-3’
Bcl-xl	F: 5’-ACTGTGCGTGGAAAGCG-3’
	R: 5’-TGGTCATTCAGATAGGTGGC-3’
NF-κB (p65)	F:5’-TCATCTTCCCGGCAGAGCCAG-3’
	R: 5’-GTGGGTCTTGGTGGTAGCTGT-3’
18S	F:5’-CGATCAGATACCGTCGTAGT-3’
	R:5’-TTCCTTTAAGTTTCAGCTTTGC-3’

### Cell migration assay

The lower chambers of cell culture inserts with 8-μm pores (NEST) were filled with 1300 µL RPMI 1640 complete medium and the upper chambers were filled with 200 µL cell suspension (1 × 10^7^/mL) resuspended in serum-free culture medium. PBS, pET-32a, and rSsAKs (10, 20, 40, 80 µg/mL) were co-incubated with the cells in the upper chamber in a 37°C 5% CO_2_ incubator for 24 h. The cells that had migrated to the lower chamber were counted with a Neubauer counting chamber.

### Detection of cytokine levels

PBS, pET-32a, and rSsAKs (10, 20, 40, 80 µg/mL) were co-cultivated with 1 mL cell suspension (1 × 10^9^/mL) in a 37°C 5% CO_2_ incubator for 24 h. The cells were mixed gently and transferred to a centrifuge tube for 5-min centrifugation at 5000 rpm to isolate the supernatant. IL-2, IL-4, IL-10, IL-17, IFN-γ, and TGF-β1 secretion was determined using enzyme-linked immunosorbent assay (ELISA) kits (Cusabio).

### Data analysis

All data were analyzed by GraphPad Prism 8.0. Differences between groups were assessed by one-way analysis of variance in SPSS 11.5. P < 0.05 was considered statistically significant.

## Results

### Identification and sequence analysis of the SsAKs

**Figure 1:**
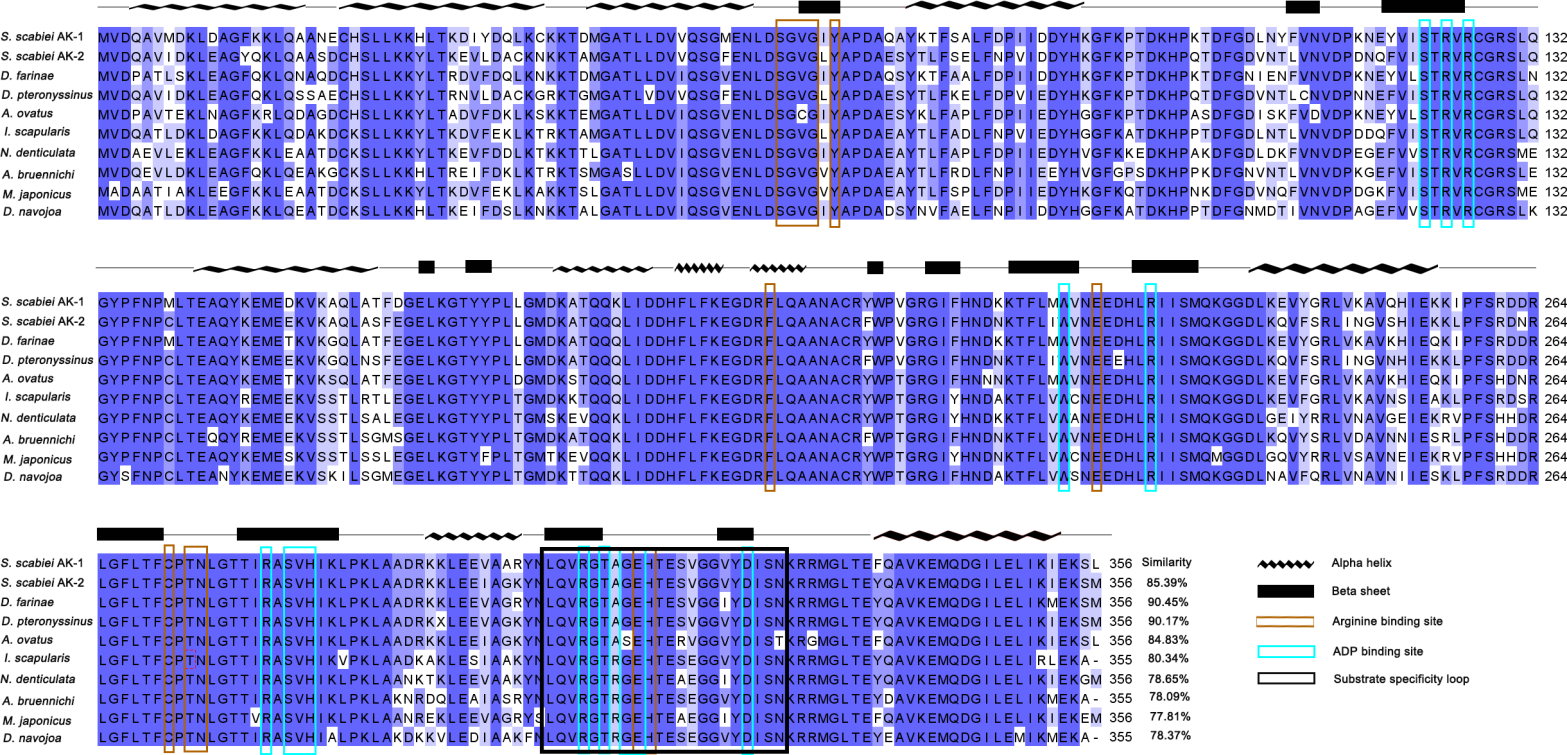
Multiple sequence alignment analysis of SsAK-1 and SsAK-2. The homology of the SsAKs to other species is shown on the right. Blue background indicates amino acids that are identical to the AKs of other species; a darker color indicates a higher consistency. Blue-green box highlights the ADP binding site. Brown box highlights the arginine binding site. The black box indicates the substrate specificity loop. The predicted secondary structure of the SsAKs is shown above the amino acid residue alignments. The AK gene accession numbers of each species are as follows: SsAK-1 (KPM07362.1), sAK-2 (OP185092), *D. farina* (AAP57094.1), *D. pteronyssinus* (ACD50950.1), *A. ovatus* (ABU97463.1), *I. scapularis* (XP_029845984.1), *Neocaridina denticulate* (BAH56609.1), *Argiope bruennichi* (KAF8790721.1), *Macrophthalmus japonicus* (AID47194.1), *Drosophila navojoa* (XP_030246642.1).

**Figure 2:**
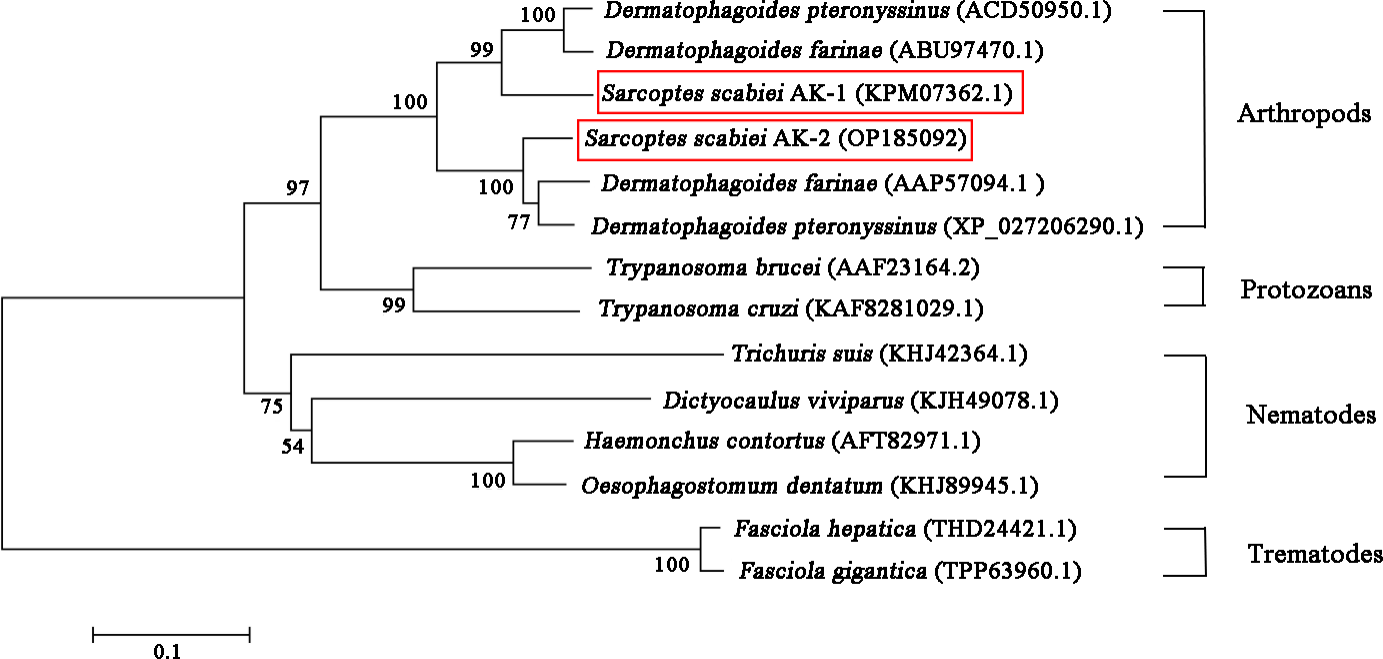
Phylogenetic relationships between SsAKs and other species. The AK amino acid sequences of 11 species were selected and the phylogenetic tree was constructed by NJ. The AK sequence accession numbers of each species are as follows: SsAK-1 (KPM07362.1), SsAK-2 (OP185092), *D. farina* (ABU97470.1), *D. pteronyssinus* (ACD50950.1), *D. pteronyssinus* (XP 027206290.1), *D. farina* (AAP57094.1), *H. contortus* (AFT82971.1), *Trichuris suis* (KHJ42364.1), *Dictyocaulus viviparus* (KJH49078.1), *Oesophagostomum dentatum* (KHJ89945.1), *Fasciola hepatica* (THD24421.1), *F. gigantica* (TPP63960.1), *T. brucei* (AAF23164.2), *T. cruzi* (KAF8281029.1).

The open reading frame showed that the SsAK-1 and SsAK-2 gene sequences contained 1,071 bp and encoded 356 amino acids with high conservation and high homology with the AK sequences of other species. The relative molecular weight of the SsAKs was 40.5 kDa and the pI was 8.36 and 6.52, respectively. The SsAKs contained no signal peptides or transmembrane regions. The SsAK-1 shared 85.39%, 90.45%, 90.17%, 84.83%, and 80.34% similarity with the AK of *Dermatophagoides farina*, *D. pteronyssinus*, *Aleuroglyphus ovatus*, and *Ixodes scapularis*, respectively. Multiple sequence alignment showed that both SsAKs had typical ADP and arginine-binding sites and substrate-specific loops (**Figure 1**). AK evolution correlates with species category, and the SsAKs were divided into two categories with high homology with *D. pteronyssinus* and *D. farina* (**Figure 2**).

### Production of rSsAKs

**Figure 3:**
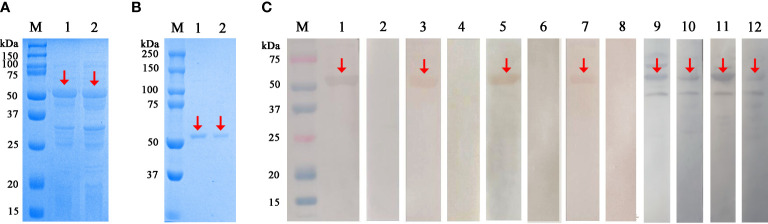
Expression, purification and western blotting analysis of the rSsAKs. **(A)** rSsAKs expressed in *E. coli* BL21 (DE3) after induction. M: Protein molecular weight marker, lane 1: rSsAK-1; lane 2: rSsAK-2. **(B)** Purified rSsAKs. M: Protein molecular weight marker, lane 1: rSsAK-1; lane 2: rSsAK-2. **(C)** Western blotting analysis of the rSsAKs. M: Protein molecular weight marker, lanes 1-2: purified rSsAK-1 detected by rat hyperimmune serum and rat negative serum, respectively; lane 3-4: purified rSsAK-1 detected by serum from rabbits naturally infected with *S. scabiei* and uninfected rabbits, respectively; lane 5-6: purified rSsAK-2 detected by rat hyperimmune serum and rat negative serum, respectively; lane 7-8: purified rSsAK-2 detected by serum from rabbits naturally infected with *S. scabiei* and uninfected rabbits, respectively; lane 9-10: rSsAK-1from total bacterial extract detected by rat hyperimmune serum and serum from rabbits naturally infected with *S. scabiei*, respectively; lane 11-12: rSsAK-2from total bacterial extract detected by rat hyperimmune serum and serum from rabbits naturally infected with *S. scabiei*, respectively. Red arrows indicate the band of interest, i.e., the rSsAKs.

The 1,071-bp SsAK-1 and SsAK-2 sequences were cloned successfully. SsAK-1 and SsAK-2 prokaryotic expression recombinant plasmids were transferred into *E. coli* BL21 cells, and rSsAK-1 and rSsAK-2 were obtained by inducing expression with 0.6 mM IPTG at the optimum temperature of 22°C. The expressed rSsAKs were ~57 kDa and contained 17-kDa tagged proteins (**Figure 3A**). The rSsAKs were purified by affinity chromatography and detected by 12% SDS-PAGE, which revealed that they were ~57 kDa (**Figure 3B**). The contents of inorganic phosphorus produced by the reaction between L-arginine and ATP catalyzed by rSsAK-1 and rSsAK-2 were 29.50 µg/mL and 29.92 µg/mL, respectively, while the catalyzation of pET-32a did not produce inorganic phosphorus, which indicated that the rSsAKs exhibited AK catalytic activity.

### Characterization of the rSsAKs

After incubation with rat hyperimmune serum or scabies mite-infected rabbit serum as the primary antibodies, the total bacterial extract and purified rSsAKs both exhibited signals at the expected protein size of ~57 kDa, which indicated that the rat and rabbit serum recognized the rSsAKs (**Figure 3C**). Incubation with healthy rat serum or healthy rabbit serum (negative control) as the primary antibodies produced no signals, which indicated that the healthy rat and healthy rabbit serum could not recognize the rSsAKs (**Figure 3C**). The results demonstrated that both rSsAKs had good immunoreactivity.

### Localization of SsAKs in mites and skin

**Figure 4:**
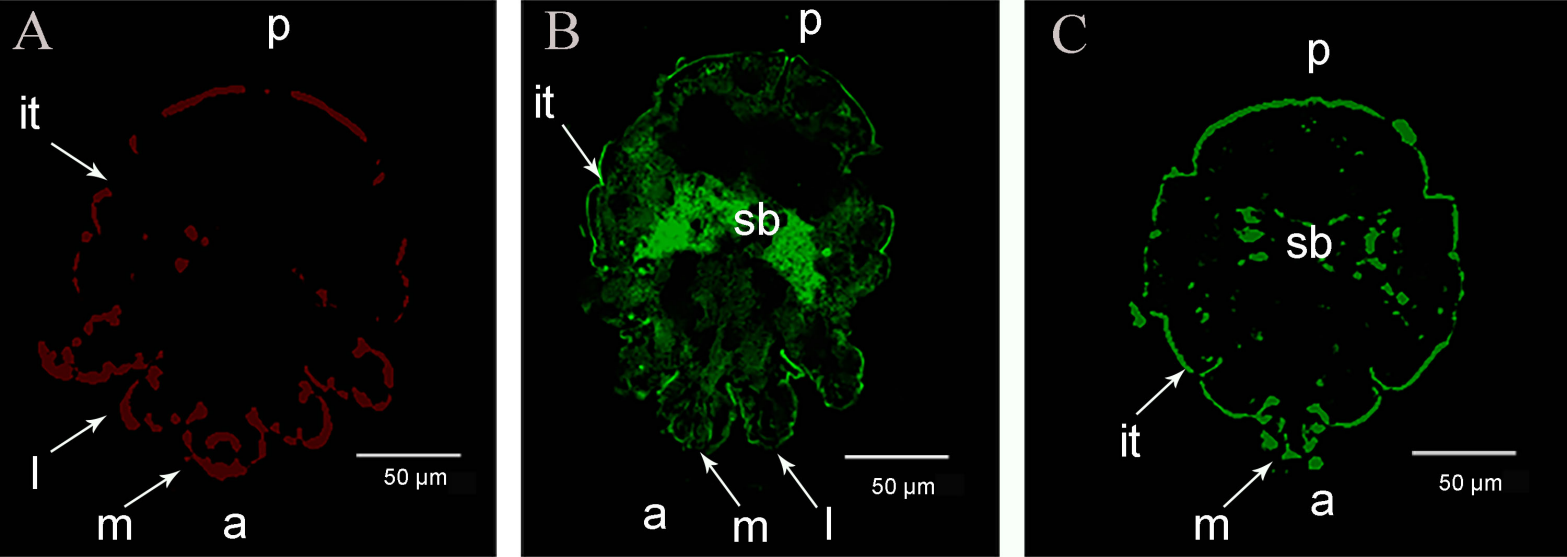
Immunolocalization of the SsAKs in *S. scabiei*. **(A)** Incubation with rat negative serum as the primary antibody. **(B)** Incubation with rat anti-rSsAK-1 IgG as the primary antibody. **(C)**. Incubation with rat anti-rSsAK-2 IgG as the primary antibody. a, front end; p, rear end; m, mouthparts; l, legs; it, integument of the exoskeleton; sb, stomach and intestine.

The immunolocalization revealed that the fluorescent signals of the SsAKs were not observed in the negative control (**Figure 4A**). The natural SsAK-1 protein was distributed in the whole body of *S. scabiei*, specifically in the exoskeleton, stomach, and intestine (**Figure 4B**). The natural SsAK-2 protein was widely distributed in the *S. scabiei* exoskeleton, chewing mouthparts, stomach, and intestine (**Figure 4C**).

**Figure 5:**
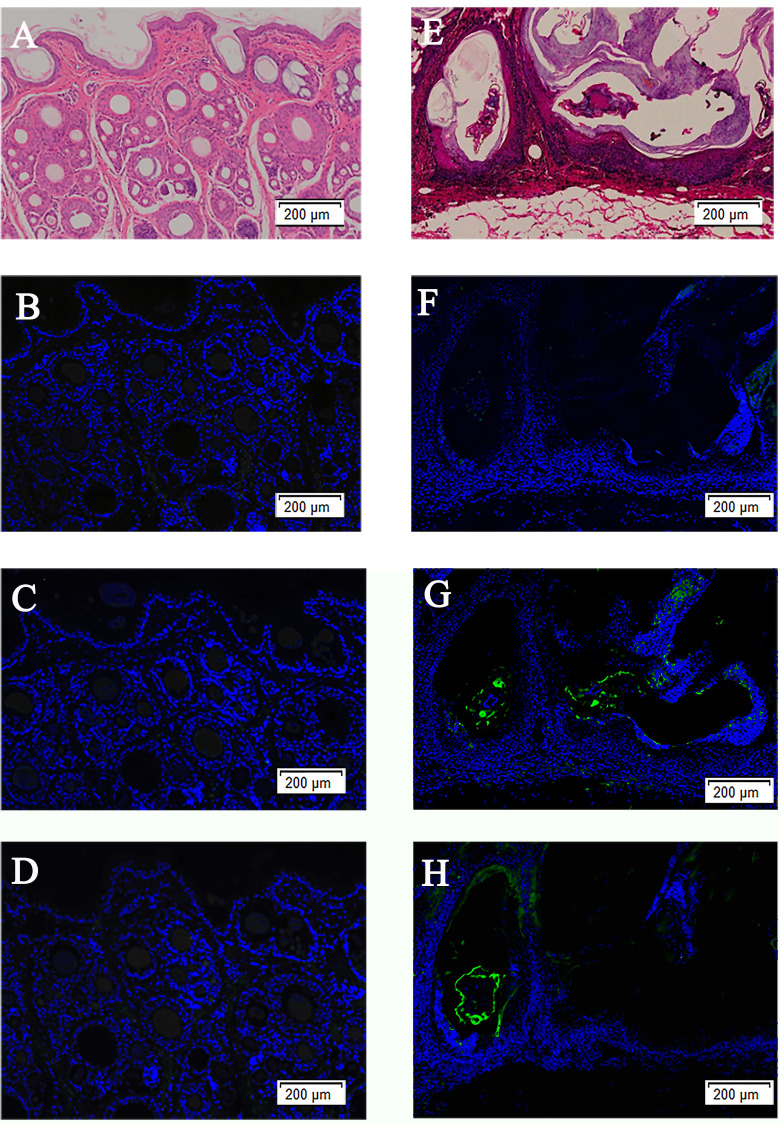
H&E staining of skin and immunolocalization of the SsAKs in the skin. H&E staining of non-skin lesions **(A)** and skin lesions **(E)** on the toes of rabbits with *S. scabiei* infection. The non-skin lesions **(B)** and skin lesions **(F)** on the toes of rabbits with *S. scabiei* incubated with rat negative serum. The non-lesion area **(C)** and skin lesion area **(G)** of the toes of rabbits with *S. scabiei* infection incubated with rat anti-rSsAK-1 IgG. The non-lesion area **(D)** and skin lesion area **(H)** of the toes of rabbits with *S. scabiei* infection incubated with rat anti-rSsAK-2 IgG. Green fluorescence: SsAK proteins.

The H&E staining and immunolocalization of healthy skin and infected skin demonstrated no obvious fluorescence signal of the SsAKs in the healthy skin and the negative control of the infected skin (**Figures 5A–F**). The natural SsAKs were both distributed in the pool around the *S. scabiei* and the epidermis (**Figures 5G, H**).

### Effects of rSsAKs on PBMC proliferation

**Figure 6:**
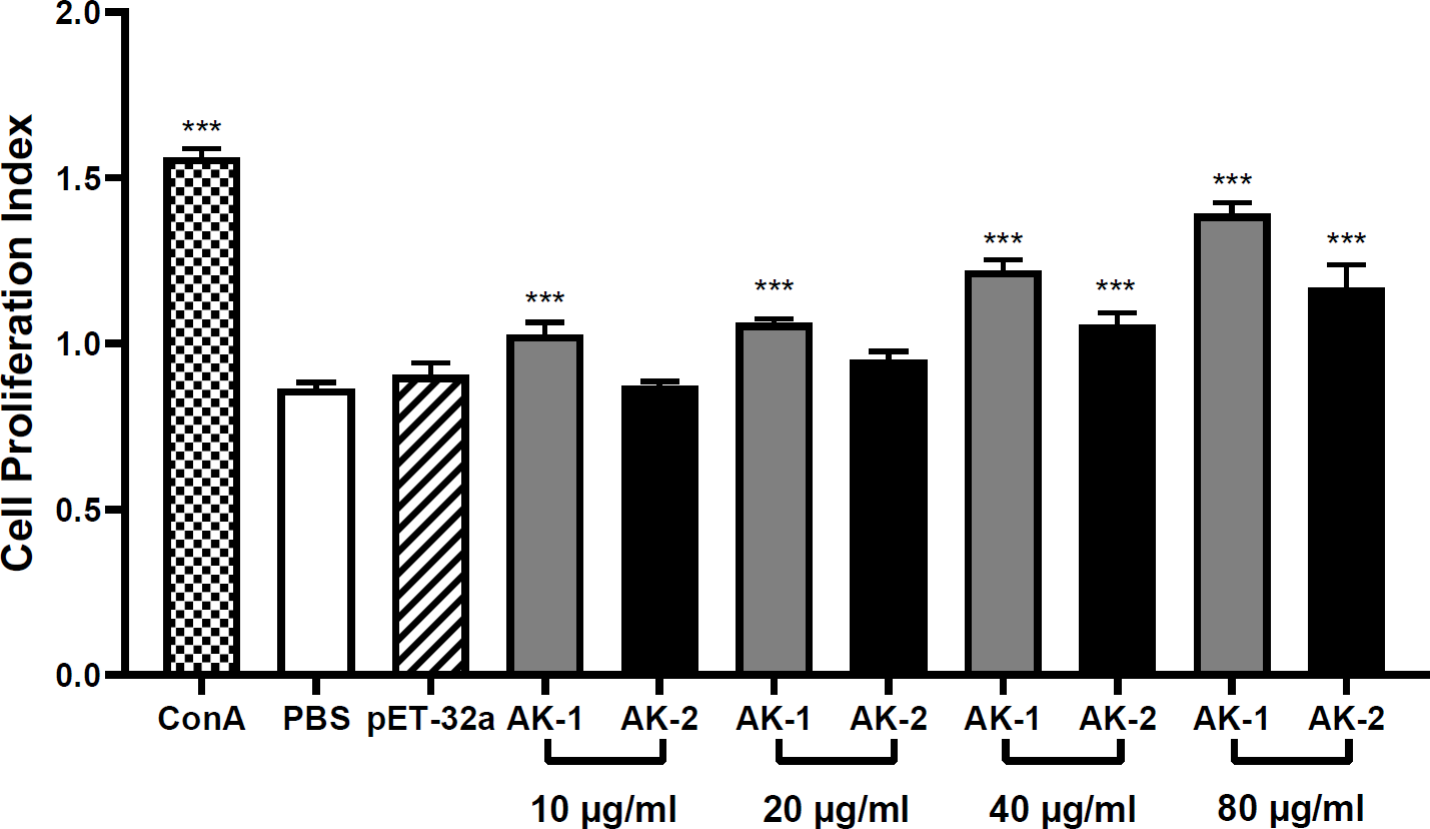
The effects of the rSsAKs on PBMC proliferation. CCK-8 detection of the effects of the rSsAKs on PBMC proliferation. The cell proliferation index was the optical density at 450 nm (OD450). Data are the mean ± SD of three replicates per group as compared to the PBS group. ***P < 0.001.

Compared with PBS, 10 μg/mL rSsAK-1 significantly increased PBMC proliferation in a concentration-dependent manner (*P* < 0.001). Compared with PBS, increased rSsAK-2 concentrations promoted PBMC proliferation activity, where 40 μg/mL rSsAK-2 increased PBMC proliferation obviously (*P* < 0.001). The influence of rSsAK-1 on PBMC proliferation was more obvious than that of rSsAK-2 (**Figure 6**).

### Effects of rSsAKs on PBMC apoptosis

**Figure 7:**
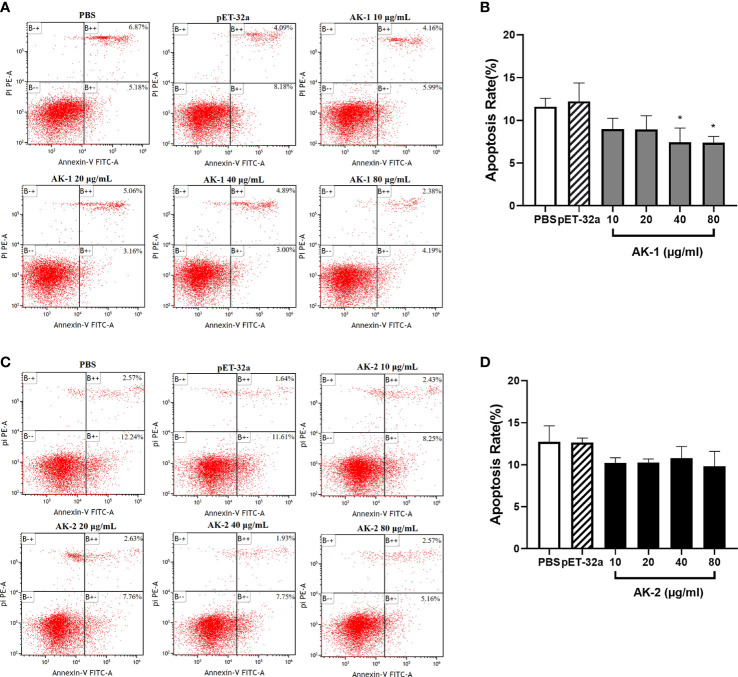
The effects of the rSsAKs on PBMC apoptosis. Annexin V/PI staining and flow cytometry detection of the effects of the rSsAKs on PBMC apoptosis. **(A, C)** Annexin V/PI staining and flow cytometry were used to detect the effects of rSsAK-1 **(A)** and rSsAK-2 **(C)** on PBMC apoptosis. **(B, D)** Changes in the total apoptosis rate of PBMCs stimulated by rSsAK-1 **(B)** and rSsAK-2 **(D)**. The total apoptosis rate was the sum of the early and late apoptosis rates. Data are the mean ± SD of three replicates per group as compared to the PBS group. *P < 0.05.

PBMC apoptosis following rSsAK stimulation was examined using flow cytometry. Annexin V–FITC+/PI phycoerythrin (PE)+ cells are late apoptotic cells and annexin V FITC+/PI PE- cells are early apoptotic cells. Compared with the PBS control, the total PBMC apoptosis rate was significantly decreased by 40 μg/mL and 80 μg/mL rSsAK-1 (*P* < 0.05) (**Figures 7A, B**). Although each rSsAK-2 concentration decreased the total PBMC apoptosis rate as compared with that of PBS, the effect was not significant (*P* > 0.05) (**Figures 7C, D**).

### The rSsAKs activated the anti-apoptotic factors and NF-κB (p65)

**Figure 8:**
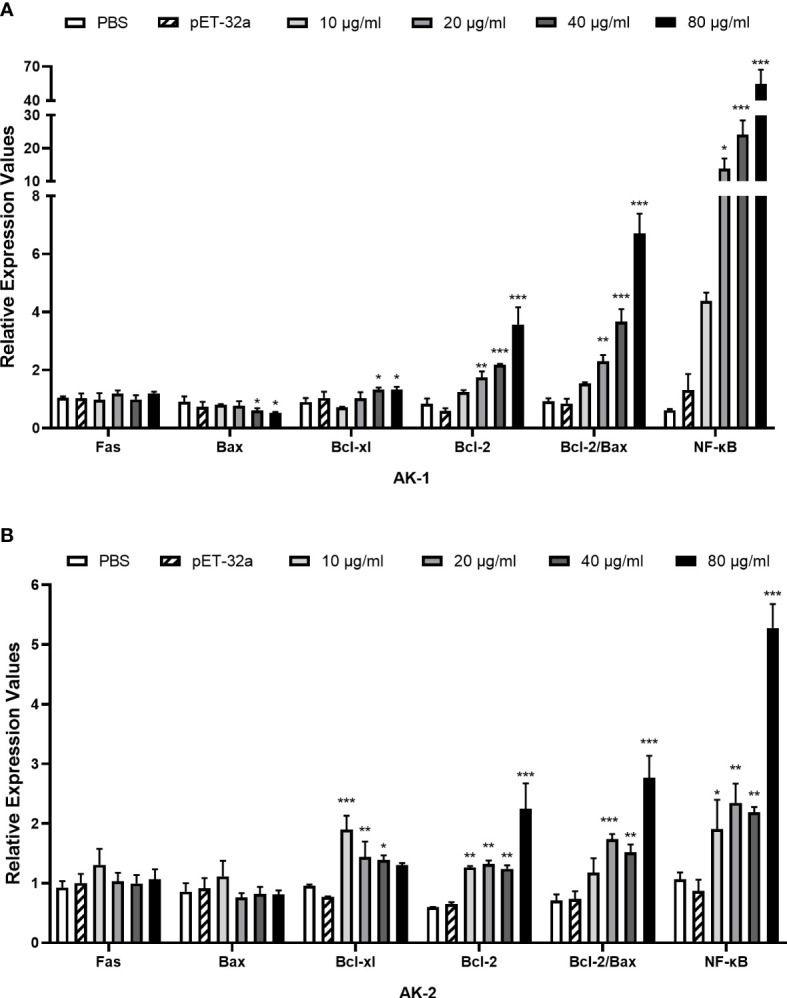
qRT-PCR results of the effects of the rSsAKs on pro-apoptotic genes, anti-apoptotic genes, and NF-κB (p65). **(A, B)** The effect of rSsAK-1 **(A)** and rSsAK-2 **(B)** on the proapoptotic genes (Fas, Bax), anti-apoptotic genes (Bcl-2, Bcl-xl), and NF-κB (p65) transcription levels. Data are the mean ± SD of three replicates per group as compared to the PBS group. *P < 0.05, **P < 0.01, ***P < 0.001.

Compared with PBS, the rSsAKs remarkably improved the transcription levels of the Bcl-2, Bcl-xl, NF-κB (p65) genes and the Bcl-2/Bax ratio in the PBMCs (*P* < 0.001) and decreased the Bax gene transcription levels (*P* < 0.05). Therefore, we speculated that the rSsAKs promoted PBMC survival by triggering the NF-κB pathway, upregulating Bcl-2 and Bcl-xl, and downregulating Bax transcription levels, but the influence of rSsAK-1 was more obvious (**Figure 8**).

### Effects of rSsAKs on PBMC migration

**Figure 9:**
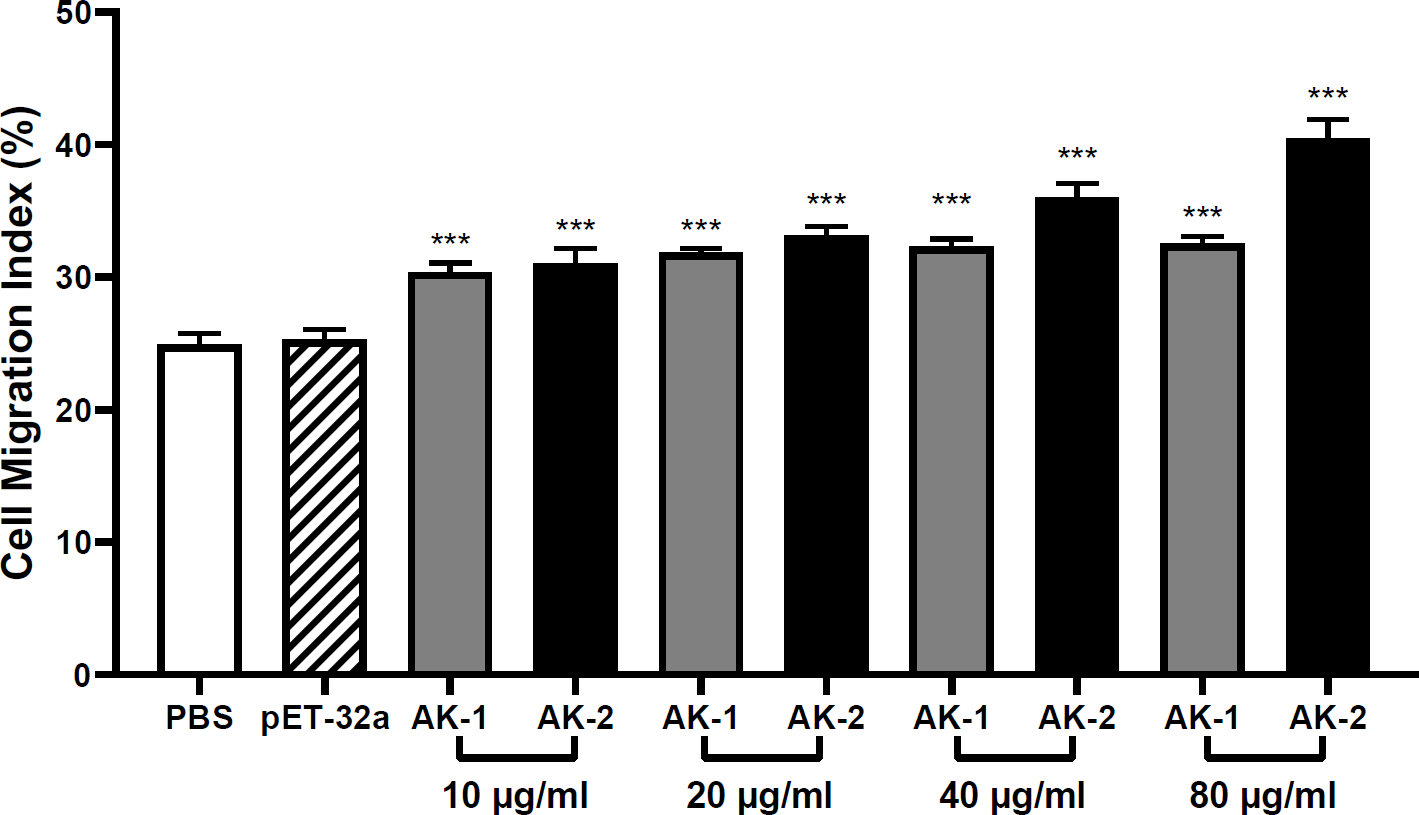
The effects of the rSsAKs on PBMC migration. The PBMC migration was counted using a Neubauer counting chamber. The migration ratio = total number of cells in the lower chamber after migration/total number of cells before migration. Data are the mean ± SD of three replicates per group as compared to the PBS group. ***P < 0.001.

Compared with the PBS control, 10 μg/mL rSsAK-1 and rSsAK-2 distinctly increased the PBMC migration rate in a concentration-dependent manner (*P* < 0.001). Therefore, we speculated that the rSsAKs promoted PBMC migration to infection sites. The effect of rSsAK-2 on PBMC migration was more obvious than that of rSsAK-1 (**Figure 9**).

### Effects of rSsAKs on PBMC cytokine secretion

**Figure 10:**
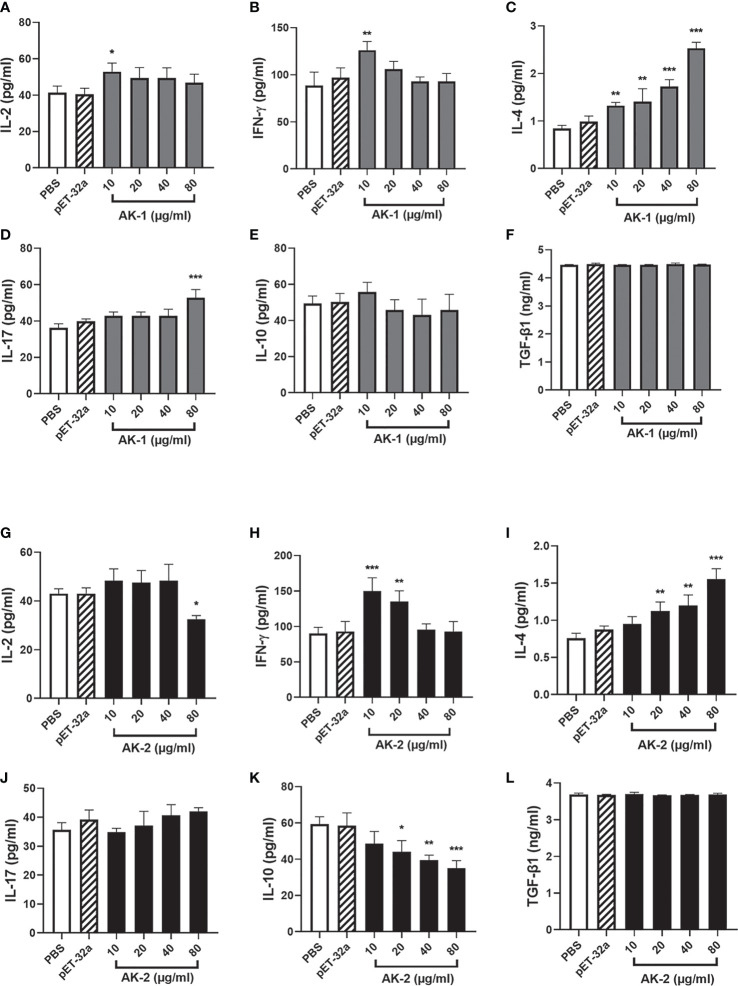
ELISA results of the effects of the rSsAKs on Th1, Th2, Th17, and Treg cytokine secretion levels. **(A-F)** The effect of rSsAK-1 on Th1 (IL-2, IFN-γ), Th2 (IL-4), Th17 (IL-17), and Treg cytokine (IL-10, TGF-β1) secretion levels. **(G-L)** The effect of rSsAK-2 on Th1 (IL-2, IFN-γ), Th2 (IL-4), Th17 (IL-17), and Treg cytokine (IL-10, TGF-β1) secretion levels. Data are the mean ± SD of three replicates per group as compared to the PBS group. *P < 0.05, **P < 0.01, ***P < 0.001.

Compared with the PBS control, low-concentration (10 μg/mL) rSsAK-1 improved IL-2 and IFN-γ secretion (*P* < 0.05). However, at 20, 40, and 80 μg/mL rSsAK-1, the IL-2 and IFN-γ levels decreased to the point where they were not significantly different from that of the PBS group (*P* > 0.05) (**Figures 10A, B**). IL-4 secretion gradually increased in a concentration-dependent manner (*P* < 0.001) (**Figure 10C**). IL-17 secretion was significantly increased only by 80 μg/mL rSsAK-1 (*P* < 0.001) and the other rSsAK-1 concentrations did not cause significant change in IL-17 secretion (*P* > 0.05) (**Figure 10D**). Furthermore, rSsAK-1 did not obviously affect IL-10 and TGF-β1 secretion (*P* > 0.05) (**Figures 10E, F**).

Compared with the PBS control, 10, 20, and 40 μg/mL rSsAK-2 did not distinctly affect IL-2 secretion (*P* > 0.05). IL-2 secretion was significantly decreased by 80 μg/mL rSsAK-2 (*P* < 0.05) (**Figure 10G**). IFN-γ secretion was significantly increased by 10 μg/mL rSsAK-2 (*P* < 0.001), but decreased in a concentration-dependent manner (**Figure 10H**). IL-4 secretion gradually increased and IL-10 secretion gradually decreased as the rSsAK-2 concentration increased (*P* < 0.001) (**Figures 10I, J**). Furthermore, rSsAK-2 stimulation had no obvious effect on IL-17 and TGF-β1 secretion (*P* > 0.05) (**Figures 10K, L**).

## Discussion

Catalyzing ATP production, AKs are mainly distributed in the metabolically active parts of the parasite. For example, *Trypanosoma brucei* AKs are localized in the flagella (15) and *Haemonchus contortus* AKs are localized in the gut (16). In the present study, immunolocalization demonstrated that SsAK-1 and SsAK-2 are mainly located in the high-energy requirement parts of *S. scabiei*, such as the exoskeleton, chewing mouthparts, legs, stomach, and intestine. Immunolocalization also confirmed that, as excretory-secretory proteins, SsAK-1 and SsAK-2 were secreted *in vitro* and existed in the pool and host epidermis, which was consistent with previous LC-MS/MS results that identified scabies mite AK as an excretory protein (11). Arthropod AKs are considered pan-allergens and immunomodulators of proinflammatory cytokines (17). AKs are allergens of *D. farina*, *T. cruzi*, and *Procambarus clarkii*, which induce Th2 polarization and allergic reactions in humans (17–19). When combined with goat PBMCs, *H. contortus* AK significantly reduced PBMC multiplication and migration and increased the apoptosis rate. The *H. contortus* AK upregulated IL-4 secretion to initiate the Th2 response that helps the host eliminate parasites; conversely, it induced Treg production to promote parasitic helminth infection (16). Clearly, the AKs participate in the parasite–host interaction to regulate host immune function.

The pathogenesis of *S. scabiei* var. *canis* may be related to the marked changes in the oxidative/antioxidant balance of peripheral blood leukocytes and the increased apoptosis rate. Dogs infected with scabies mites were in a state of marked oxidative stress with altered antioxidant defense mechanisms, likely due to excess reactive oxygen radical production from inflammatory cells recruited to fight the mites, which led to antioxidant system exhaustion and increased peripheral blood leukocyte apoptosis, enabling the mites to escape the host immune defense easily to multiply (20). Apoptosis is mainly regulated by the extrinsic apoptotic pathway and the intrinsic mitochondria-dependent pathway, and involves the B cell lymphoma 2 family members, which consist of anti-apoptotic factors (Bcl-2, Bcl-xl) and pro-apoptotic factors (Bax) (21–23). Here, the rSsAKs increased Bcl-2 and Bcl-xl transcription levels and the Bcl-2/Bax ratio, which indicated that the rSsAKs promoted PBMC proliferation and inhibited PBMC apoptosis, thereby promoting cell survival and limiting *S. scabiei* proliferation in early infection (23). Furthermore, both rSsAK-1 and rSsAK-2 increased NF-κB (p65) transcription levels, particularly rSsAK-1. NF-κB signaling participates in cell survival (24) and RelA/p65 is the most important subunit in the NF-κB family (25). It may indicate that PBMC survival is achieved by triggering the NF-κB pathway, resulting in enhanced Bcl-2 and Bcl-xl expression (26, 27). Bcl-xl expression is first induced by the NF-κB canonical pathway, then enhanced and stabilized by the non-canonical pathway. The transcription level of Bcl-xl reduced with the improvement of rSsAK-2, probably due to the fact that rSsAK-2 did not trigger the non-canonical pathway so that could not maintain its levels (28).

Immune cell migration and chemotaxis play vital roles in the inflammatory process (29). For example, the gradual migration of circulating lymphocytes and monocytes into the lungs in patients with chronic obstructive pulmonary disease leads to the development of chronic inflammation (30). Here, both rSsAK-1 and rSsAK-2 promoted PBMC migration, and high concentrations of rSsAK-2 exerted a particularly significant effect, inducing PBMC migration into the skin lesions and participating in the course of local infection. PBMC migration is regulated by many chemokines and adhesion factors (31, 32), which in turn are regulated by cytokines (33). Here, the rSsAKs promoted IL-4 secretion while rSsAK-2 inhibited IL-10 secretion. IL-10 inhibits the secretion of most chemokines (CXCL10, CXCL11, CCL22) that participate in establishing the inflammatory environment, thereby limiting monocyte migration and their ability to recruit inflammatory cells (34, 35). IL-4 also influences immune cell migration to allergic inflamed tissues (36) and increases CCL22 expression to recruit lymphocytes into the inflamed epidermis (32, 37). This causes more PBMCs and inflammatory cells to aggregate at the infection site, and the cytokines they secrete in turn prompt more inflammatory cells (eosinophils, mastocytes, basophils) to participate in local infection, which leads to IgE antibody production to exacerbate inflammation (38, 39).

The parasite-triggered host immune response mainly includes Th1/Th2 and Th17/Treg. Under normal conditions, the Th1 and Th2 responses balance each other to establish a steady state (40), and an imbalance between the two leads to non-protective and dysfunctional allergic reactions (41). Allergy is an immune dysregulation caused by an imbalance between Th2, Treg, and Th17 (42, 43). The Th17/Treg balance is crucial in human autoimmune and inflammatory diseases and is a major player in autoimmunity (44–46). IL-4 is the main factor that causes Th2 polarization (47), and IFN-γ promotes Th1 cytokine secretion (48). Mainly secreted by Th17 cells, IL-17 is an effective proinflammatory cytokine while the anti-inflammatory cytokine IL-10 is primarily produced by Treg cells (49, 50). In the present study, the increase in the rSsAK concentrations was followed by the gradual decrease in the secretion of Th1 cytokines (IL-2, IFN-γ), while IL-4 secretion levels gradually increased, which indicated that the Th1 response was gradually biased towards the Th2 response, causing a Th1/Th2 imbalance (51–53). The IL-17 secretion level increased upon rSsAK-1 stimulation and that of IL-10 decreased when incubated with rSsAK-2, suggesting a predominant Th17 response. As the disease course develops, the IL-10 function of inhibiting Th2 cells and downregulating eosinophils is weakened, which is conducive to Th2 polarization (54). Upon *S. scabiei* infection, the host immune response is mainly driven by a hypersensitivity reaction partly caused by Th2 cytokine release, which induces increased vascular permeability and allergic inflammation (55–57). AK is also allergen 20 of *D. farinae*, namely Der f 20, which induced asthma, Th2 polarization, and allergic reactions in mice (18). A shrimp allergen, AK stimulates human PBMCs to generate specific T cell lines and upregulate Th2 and Th17 cytokine levels (19). Th2 polarization induced by *S. scabiei* AKs may be an important cause of host allergy. Furthermore, when *S. scabiei* extract stimulates human PBMCs, Treg function and IL-10 production are upregulated, which is possibly part of the reason for the delayed appearance of initial symptoms of *S. scabiei* infection and immunosuppression (58). On the contrary, rSsAK-2 caused decreased IL-10 secretion, which was beneficial for improving host immunity, accelerating the appearance of local symptoms, and resisting *S. scabiei* parasitism.

The NF-κB pathway is crucial to T cell and Treg production and homeostasis (59) and is involved in immune regulation, inflammation, and other processes (60–62). The main allergens of *D. pteronyssinus* activated NF-κB to increase eosinophil numbers and exacerbate allergic inflammation (63). In *Psoroptes ovis* infection, NF-κB regulated the production of the main mediators of the inflammatory response, namely a series of cytokines, chemokines, selectins, and integrins that have vital impacts on the proinflammatory response (64). Our results demonstrated that both rSsAK-1 and rSsAK-2 induced elevated transcription levels of NF-κB (p65). Therefore, the NF-κB pathway might be vital for modifying the allergic inflammation induced by *S. scabiei* AKs (65).

In this study, the results revealed the roles of rSsAKs in promoting the development of allergic inflammation through in-vitro experiments, among which rSsAK-1 is mainly focused on promoting cell survival and Th2 polarization, while rSsAK-2 is mainly focused on inducing cell migration to the infection site. Generally speaking, in-vivo studies were needed to validate the finding of the in-vitro study. In previous studies, in-vitro experiments showed that Der f 20 and Der p1 from dust mites mainly function in promoting the release of pro-inflammatory cytokines of dendritic cells and epithelial cells, and the in-vivo experiments revealed similar functions of promoting the appearance of inflammatory symptoms, such as airway hyperresponsiveness and inflammation in lung tissue (66, 67). These indicated that the results of in-vitro and in-vivo experiments are consistent. Our research group mainly focus on screening genes and proteins in *S.scabiei* that have important roles in inducing inflammation and allergy, the ultimate goal is to develop new therapeutics or vaccines for the control of scabies. To achieve this goal, in-vitro experiments were used to screen proteins preliminarily, and in the future, we plant to use parallel in-vivo experiments to validate the function of these proteins and obtain proteins that play vital roles in host-parasite immune interplay.

## Conclusion

In *S. scabiei*, SsAK-1 and SsAK-2 are mainly located in the parts with high energy requirements, such as the exoskeleton, chewing mouthparts, legs, stomach, and intestine, and can be secreted in the pool and epidermis of the skin lesions to regulate the host immunity reaction. rSsAK-1 and rSsAK-2 obviously improved the transcription levels of NF-κB (p65) and anti-apoptotic factors to promote host PBMC proliferation, inhibit PBMC apoptosis, and induce PBMC migration to the infected areas. Furthermore, rSsAK-1 and rSsAK-2 shifted the Th1/Th2 balance toward Th2 and changed the Th17/Treg balance, suggesting that they may promote the development of allergic inflammation. This study provided evidence for the pathogenic mechanism of *S. scabiei* infection in humans and animals and a basic reference for the exploitation of novel medicine and vaccines in the future.

## Data availability statement

The original contributions presented in the study are included in the article/supplementary material, further inquiries can be directed to the corresponding author.

## Ethics statement

This animal study was reviewed and approved by the Sichuan Agricultural University Animal Ethics Committee.

## Author contributions

YaX participated in designing and performing the experiments, feeding the experimental animals, statistical analysis, and manuscript writing. YaX and ZX participated in animal feeding and the experiments. XG, YuX, RH, JX, BJ, and XP contributed to the sample collection and performing the experiments. GY participated in designing the study. XG, YuX, RH, and JX assisted in designing the study. All authors contributed to the article and approved the submitted version

## Funding

This work was supported by grants from the Natural Science Foundation of Sichuan Province (Grant No. 2022NSFSC1658) and China Postdoctoral Science Foundation (Grant No. 2020M683652XB).

## Acknowledgments

The authors are extremely grateful to the teachers and classmates at the Public Laboratory of Sichuan Agricultural University in Sichuan Province for kindly allowing us to carry out the relevant experiments. We also thank the native English-speaking scientists of Elixigen (Huntington Beach, CA) for editing our manuscript.

## Conflict of interest

The authors declare that they do not have any commercial or associative interest that represents a conflict of interest in connection with the work submitted.

## Publisher’s note

All claims expressed in this article are solely those of the authors and do not necessarily represent those of their affiliated organizations, or those of the publisher, the editors and the reviewers. Any product that may be evaluated in this article, or claim that may be made by its manufacturer, is not guaranteed or endorsed by the publisher.
